# Supplementary material for: Novel Driver Strength Index highlights important cancer genes in TCGA PanCanAtlas patients
Source: PeerJ. 2022 Aug 11;10:e13860. doi: 10.7717/peerj.13860 (PMC9375969; doi:10.7717/peerj.13860)
Supplement: Supplemental Information 2 [file peerj-10-13860-s002.zip › COHORTS/genes plots/age/2021_8_16_14_9_distribution_cohorts_males_30-34.pdf]

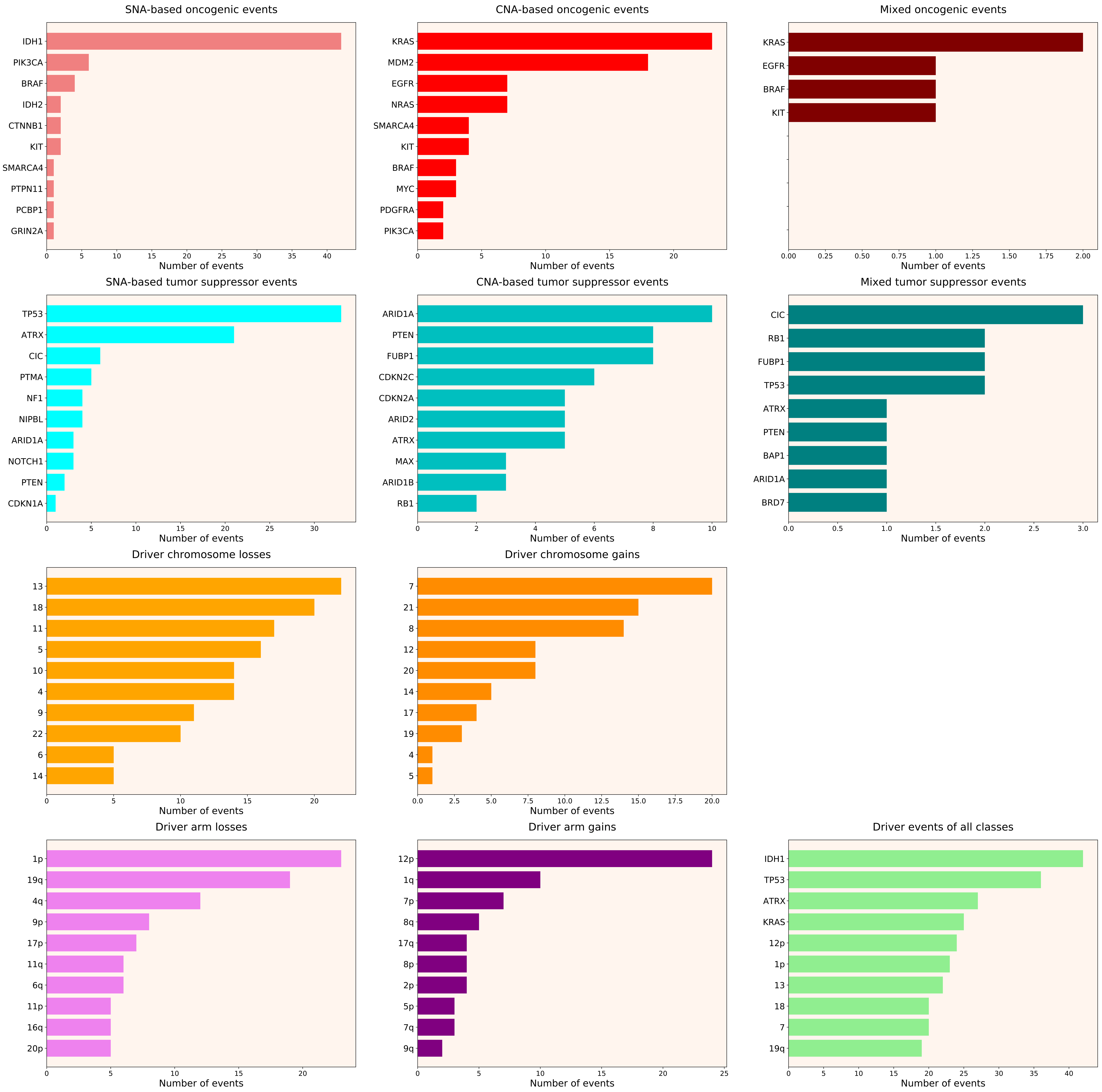

SNA-based oncogenic events
IDH1
PIK3CA
BRAF
IDH2
CTNNB1
KIT
SMARCA4
PTPN11
PCBP1
GRIN2A
Number of events
CNA-based oncogenic events
KRAS
MDM2
EGFR
NRAS
SMARCA4
KIT
BRAF
MYC
PDGFRA
PIK3CA
Number of events
Mixed oncogenic events
KRAS
EGFR
BRAF
KIT
Number of events
SNA-based tumor suppressor events
TP53
ATRX
CIC
PTMA
NF1
NIPBL
ARID1A
NOTCH1
PTEN
CDKN1A
Number of events
CNA-based tumor suppressor events
ARID1A
PTEN
FUBP1
CDKN2C
CDKN2A
ARID2
ATRX
MAX
ARID1B
RB1
Number of events
Mixed tumor suppressor events
CIC
RB1
FUBP1
TP53
ATRX
PTEN
BAP1
ARID1A
BRD7
Number of events
Driver chromosome losses
13
18
11
5
10
4
9
22
6
14
Number of events
Driver chromosome gains
7
21
8
12
20
14
17
19
4
5
Number of events
Driver arm losses
1p
19q
4q
9p
17p
11q
6q
11p
16q
20p
Number of events
Driver arm gains
12p
1q
7p
8q
17q
8p
2p
5p
7q
9q
Number of events
Driver events of all classes
IDH1
TP53
ATRX
KRAS
12p
1p
13
18
7
19q
Number of events
